# Retrospective study: risk assessment model for osteoporosis—a detailed exploration involving 4,552 Shanghai dwellers

**DOI:** 10.7717/peerj.16017

**Published:** 2023-09-08

**Authors:** Dan Han, Zhongcheng Fan, Yi-sheng Chen, Zichao Xue, Zhenwei Yang, Danping Liu, Rong Zhou, Hong Yuan

**Affiliations:** 1 Department of Emergency Medicine and Intensive Care, Songjiang Hospital Affiliated to Shanghai Jiaotong University School of Medicine (Preparatory Stage), Shanghai, Shanghai, China; 2 Department of Orthopaedics, Hainan Province Clinical Medical Center, Haikou Orthopedic and Diabetes Hospital of Shanghai Sixth People’s Hospital, Haikou, China; 3 Department of Sports medicine, Huashan Hospital, Fudan University, Shanghai, China; 4 Department of Orthopaedics, Qingdao Hospital, University of Health and Rehabilitation Sciences (Qingdao Municipal Hospital), Qingdao, China; 5 Department of Orthopaedics, First Affiliated Hospital of Jinzhou Medical University, Jinzhou, China; 6 Department Two of Medical Administration, Zhongshan Hospital, Fudan University, Shanghai, China

**Keywords:** Osteoporosis, Bone loss, Clinical prediction model, Retrospective study

## Abstract

**Background:**

Osteoporosis, a prevalent orthopedic issue, significantly influences patients’ quality of life and results in considerable financial burden. The objective of this study was to develop and validate a clinical prediction model for osteoporosis risk, utilizing computer algorithms and demographic data.

**Method:**

In this research, a total of 4,552 residents from Shanghai were retrospectively included. LASSO regression analysis was executed on the sample’s basic characteristics, and logistic regression was employed for analyzing clinical characteristics and building a predictive model. The model’s diagnostic capacity for predicting osteoporosis risk was assessed using R software and computer algorithms.

**Results:**

The predictive nomogram model for bone loss risk, derived from the LASSO analysis, comprised factors including BMI, TC, TG, HDL, Gender, Age, Education, Income, Sleep, Alcohol Consumption, and Diabetes. The nomogram prediction model demonstrated impressive discriminative capability, with a C-index of 0.908 (training set), 0.908 (validation set), and 0.910 (entire cohort). The area under the ROC curve (AUC) of the model was 0.909 (training set), 0.903 (validation set), and applicable to the entire cohort. The decision curve analysis further corroborated that the model could efficiently predict the risk of bone loss in patients.

**Conclusion:**

The nomogram, based on essential demographic and health factors (Body Mass Index, Total Cholesterol, Triglycerides, High-Density Lipoprotein, Gender, Age, Education, Income, Sleep, Alcohol Consumption, and Diabetes), offered accurate predictions for the risk of bone loss within the studied population.

## Introduction

Osteoporosis, a prevalent orthopedic disorder, frequently results in fractures, considerably impacting patients’ quality of life and escalating financial burdens (Leslie & Morin, 2014). The primary cause of osteoporotic fractures is a decline in bone mass and density due to various factors, leading to decreased elasticity and increased brittleness (Leslie & Morin, 2014). Over the past decade, the incidence of osteoporosis has surged, affecting more than a third of individuals aged 50 and above. The current clinical treatments predominantly comprise anti-bone resorption drugs, bone formation stimulants, and certain herbal remedies. Nevertheless, their efficacy remains suboptimal (Zhang et al., 2016; Khosla & Hofbauer, 2017). Although surgical procedures are an option, they entail numerous postoperative complications (Russell, 2013).

Detailed insight into the status of osteoporosis prevention and treatment, elaborating on various preventive measures, pharmacological interventions, and lifestyle changes, provides a better understanding of the backdrop of our study. This information helps shed light on the nuances of osteoporosis management and the research focus in this area (Kerschan-Schindl, 2016). Consequently, managing osteoporosis from an etiological prevention perspective may be an effective strategy to avert and treat osteoporosis and related disorders in the future (Kerschan-Schindl, 2016). Health management involves an exhaustive analysis, detection, prediction, evaluation, prevention, and maintenance of health risk factors for healthy individuals, those with suboptimal health, and patient groups. The overarching objective of health management is to transition from passive disease treatment to proactive health management, ultimately conserving medical expenses and promoting overall health.

Bone mineral density (BMD) remains the gold standard for assessing bone mass and diagnosing osteoporosis. Early prediction of bone loss can aid in preventing osteoporosis onset, a crucial factor for enhancing patients’ quality of life (Lane, 2006; Tella & Gallagher, 2014; Chen et al., 2020b). Several factors influence bone loss in the population, including age, obesity, physical activity, occupation type, lifestyle, and environment. However, these conventional methods lack the precision required to predict the risk of bone loss in the population accurately. An expanded literature review for our study includes the latest research and relevant findings in the field of osteoporosis prevention and treatment, emphasizing the ability of nomogram prediction models to predict disease risk from an etiological perspective (Chen et al., 2020a; Kang et al., 2020; Liu & Li, 2021; Zagórski et al., 2021).

Consequently, promoting bone health would be advantageous if the risk of bone loss in a population could be effectively and effortlessly predicted. The central focus of this study was the development of a nomogram prediction model, a tool that has been increasingly adopted in medical research and patient care for its utility in predicting clinical outcomes (Liang et al., 2015; Li et al., 2021). A nomogram is a graphical representation that combines multiple variables to estimate the probability of a particular outcome or event. It can help healthcare professionals make wise decisions about patient care based on an individual’s specific characteristics (Iasonos et al., 2008).

In our study, a nomogram prediction model was crafted based on the fundamental characteristics of the population. The model’s diagnostic performance in predicting the risk of developing bone loss was assessed using a computer algorithm. With the assistance of the nomogram, we were able to provide a quantitative tool to estimate the risk of bone loss, enhancing the understanding of osteoporosis and aiding in early diagnosis and personalized intervention strategies. The introduction of the nomogram in this study contributes to the existing body of knowledge by providing a more precise and individualized assessment of osteoporosis risk.

## Materials and Methods

### Inclusion of participants and data collection

The study population was primarily sourced from individuals aged 18 to 89 years who attended physical examinations and consultations in Shanghai, China from January 1st, 2019, to January 18th, 2023. We initiated a comprehensive participant recruitment process, delineating specific inclusion and exclusion criteria for the study. Participants with acute and chronic liver and kidney diseases, endocrine diseases, and those taking long-term medications affecting bone metabolism were excluded, ensuring a homogenous study population with minimal confounding factors. Our enrollment procedure was systematically structured, with careful documentation of participant details and consent. The study received approval from the local ethics committee of Songjiang Hospital Affiliated to Shanghai Jiaotong University School of Medicine (Preparatory Stage) (Approval number 2023SQ001), reinforcing the ethical conduct of the research. Every participant was briefed about the study and signed an informed consent form, facilitating transparency and ethical adherence. To understand our study population better, we collected extensive information, encompassing their general conditions and lifestyle habits. This included data on demographic characteristics such as gender, marital status, education level, and income. Furthermore, lifestyle habits and medical conditions were documented, such as smoking habits, diet, sleep, alcohol consumption, hypertension (HPT), diabetes (DBT), and hyperlipidemia (HLP). Biochemical measurements included low-density lipoprotein (LDL), total cholesterol (TC), fasting blood glucose (FBG), and high-density lipoprotein (HDL). Information on assessing bone mineral density, biochemical markers, and other relevant tests was carefully recorded. Our data collection process was meticulously described, detailing our data sources, the inclusion and exclusion criteria of the study population, and the procedure for data extraction and transformation. Measures to ensure data quality, such as using standardized data collection forms, double data entry, and validation checks, were implemented to identify and correct any inconsistencies or differences.

### Diagnostic criteria for decreased bone mass

The diagnosis of osteoporosis adheres to the globally recognized World Health Organization (WHO) criteria (Kanis & Kanis, 1994; Kanis et al., 2009). Bone mineral density (BMD) was measured on the distal 1/3 of the ulnar radius of the non-stressed side of the forearm using dual-energy X-ray absorptiometry (DXA) with a GE Lunar iDXA densitometer (GE Healthcare, WI, USA) (Kanis et al., 2009). This specific site for BMD measurement was chosen primarily due to certain limitations and contraindications in measuring lumbar spine and hip in our study population. However, we acknowledge concerns regarding the wide acceptance of traditional DXA examination sites such as lumbar spine, hip, and the upper one-third of the femur. Following the diagnostic categorization proposed by Kanis et al. (2009) and the WHO, individuals with T-scores ≥ −1.0 were considered to have normal bone mass, while those with T-scores <−1.0 were classified as having reduced bone mass. T ≤−2.5 is defined as applicable to patients with osteoporosis. Our study adhered to these standards and definitions to maintain rigorous diagnostic accuracy and comparability of our results.

### Statistical analysis, predictive model building and validation

We utilized R software (version 3.5.3; R Core Team, 2019) for data processing, and statistical significance was considered at *P* values <0.05. Categorical data were expressed as the number of cases and percentages, and compared between groups using chi-square test. Non-normally distributed measures were expressed as median(quartiles) [M(Q_L_, Q_U_)], and the rank sum test was used for group comparisons. We utilized the LASSO regression analysis, a statistical method used for variable selection and regularization, using the “glmnet” package to identify potential predictors associated with the risk of bone loss. Based on these predictors, patients were divided into testing and training groups, with the occurrence of bone loss serving as the dependent variable. A logistic regression analysis was then performed to elucidate the risk factors associated with bone loss. We selected predictors for our nomogram based on their statistical significance in multivariate analysis and their clinical relevance and utility. Nomograms were then constructed based on these findings (Iasonos et al., 2008). To assess the validity and predictive performance of the nomogram, the Bootstrap resampling method was subsequently employed. The predictive accuracy of the model was quantified using the concordance index (C-index), with a value closer to 1 indicating higher accuracy. Receiver operating characteristic(ROC) curves were plotted to further evaluate the discriminatory power of each predictor in determining the risk of bone loss. Additionally, decision curve analysis was conducted using the “rmda” package to evaluate the clinical utility of the model in predicting the risk of bone loss (Yoo et al., 2013). Continuous variables are presented as means ± SEM. A two-tailed unpaired student *t*-test was used for comparing two groups; and for multiple groups, we applied the one-way ANOVA followed by post-hoc tests (Tukey’s HSD) for determining specific differences between individual groups.

## Results

### Detailed demographics and clinical characteristics of the study population

Our patient cohort, encompassing 4,552 individuals, consisted of 517 diagnosed with osteopenia and a further subset of 92 suffering from osteoporosis. The cohort’s gender distribution included 2,171 males and 2,381 females. Table 1 offers an exhaustive demographic and clinical delineation of the participants, classified according to their bone health status into: ‘No Osteopenia’, ‘Osteopenia’, and ‘Osteoporosis’. The table unfolds an intricate portrait of patient characteristics, encapsulating age, gender distribution, marital status, educational background, income bracket, lifestyle habits, and prevailing comorbidities. Further, metabolic indicators, including levels of low-density lipoprotein (LDL), total cholesterol (TC), triglycerides (TG), fasting blood glucose (FBG), and high-density lipoprotein (HDL) are meticulously charted. Moreover, an interdependence matrix is furnished as Fig. S1 to elucidate the correlations amidst these characteristics. The total cohort was subsequently segregated at a 7:3 ratio into a training subset, encompassing 3187 cases, and a validation subset with 1,365 cases. Table 2 delineates the fundamental clinical characteristics of patients in the training and validation sets. These attributes encompass age, gender, marital status, educational attainment, income, smoking habits, salt intake, sleep duration, alcohol consumption, hypertension, diabetes, hyperlipidemia, and several blood biochemical indicators such as low-density lipoprotein (LDL), body mass index (BMI), Total cholesterol (TC), Triglycerides (TG), Fasting Blood Glucose (FBG), and high-density lipoprotein (HDL). Each characteristic is accompanied by its respective distribution and proportion within the normal group, osteoporosis group, and osteopenia group. Moreover, for each attribute, *p*-values are furnished to demonstrate the statistical discrepancies among the groups. To elaborate, regarding age (median, interquartile range), within the training and validation sets, the median age for the normal group consistently stands at 38, whereas the osteoporosis group’s median age is recorded at 64 and 66, respectively. The distribution of traits such as gender, marital status, educational level, income, smoking habits, alcohol consumption, hypertension, diabetes, and hyperlipidemia are lucidly presented as well.

**Table 1:** Basic clinical characteristics of patients.

Characteristic	No Osteopenia [*n* = 3943]	Osteopenia [*n* = 517]	Osteoporosis [*n* = 92]	*p* value
Age (year), meidan (IQR)	38 (33, 50)	63 (55, 67)	64 (60.75, 67)	<0.001
Gender				<0.001
FEMALE	1904 (41.8%)	389 (8.5%)	88 (1.9%)	
MALE	2039 (44.8%)	128 (2.8%)	4 (0.1%)	
Marriage				<0.001
Divorced	25 (0.5%)	3 (0.1%)	0 (0%)	
Married	3745 (82.3%)	501 (11%)	90 (2%)	
Remarry	4 (0.1%)	1 (0%)	0 (0%)	
Spinsterhood	160 (3.5%)	1 (0%)	0 (0%)	
Widowed	9 (0.2%)	11 (0.2%)	2 (0%)	
Education				<0.001
Illiteracy	21 (0.5%)	17 (0.4%)	7 (0.2%)	
Junior high school	397 (8.7%)	165 (3.6%)	27 (0.6%)	
Primary school	79 (1.7%)	63 (1.4%)	14 (0.3%)	
Senior high school	582 (12.8%)	152 (3.3%)	32 (0.7%)	
Higher education	2864 (62.9%)	120 (2.6%)	12 (0.3%)	
Income (yuan)				<0.001
<1000	7 (0.2%)	13 (0.3%)	2 (0%)	
1000–3000	293 (6.4%)	125 (2.7%)	34 (0.7%)	
3000–5000	3496 (76.8%)	360 (7.9%)	53 (1.2%)	
5000–10000	123 (2.7%)	17 (0.4%)	2 (0%)	
>10000	24 (0.5%)	2 (0%)	1 (0%)	
Smoke				<0.001
NO	2969 (65.2%)	440 (9.7%)	88 (1.9%)	
Quitting	118 (2.6%)	14 (0.3%)	0 (0%)	
YES	856 (18.8%)	63 (1.4%)	4 (0.1%)	
Dietary salt intake(g)				0.060
<6	1120 (24.6%)	167 (3.7%)	30 (0.7%)	
6–12	2059 (45.2%)	270 (5.9%)	51 (1.1%)	
>12	764 (16.8%)	80 (1.8%)	11 (0.2%)	
Sleep (hour)				<0.001
<4	22 (0.5%)	15 (0.3%)	3 (0.1%)	
4–5.9	332 (7.3%)	109 (2.4%)	22 (0.5%)	
6–6.9	1176 (25.8%)	200 (4.4%)	32 (0.7%)	
7–7.9	1894 (41.6%)	159 (3.5%)	28 (0.6%)	
8–9.9	504 (11.1%)	30 (0.7%)	7 (0.2%)	
>10	15 (0.3%)	4 (0.1%)	0 (0%)	
Drink				<0.001
NO	3325 (73%)	469 (10.3%)	90 (2%)	
YES	618 (13.6%)	48 (1.1%)	2 (0%)	
HPT				<0.001
NO	2960 (65%)	294 (6.5%)	56 (1.2%)	
YES	983 (21.6%)	223 (4.9%)	36 (0.8%)	
DBT				<0.001
NO	3796 (83.4%)	457 (10%)	85 (1.9%)	
YES	147 (3.2%)	60 (1.3%)	7 (0.2%)	
HLP				<0.001
NO	2157 (47.4%)	229 (5%)	35 (0.8%)	
YES	1786 (39.2%)	288 (6.3%)	57 (1.3%)	
LDL (mmol/L), meidan (IQR)	2.05 (1.59, 2.54)	2.33 (1.89, 2.83)	2.41 (1.84, 3.01)	<0.001
BMI (kg/m^2^), meidan (IQR)	24.2 (22, 26.6)	23.6 (21.8, 25.7)	22.9 (21.5, 25)	<0.001
TC (mmol/l), meidan (IQR)	4.83 (4.27, 5.46)	5.28 (4.66, 5.97)	5.31 (4.8, 6.04)	<0.001
TG (mmol/l), meidan (IQR)	1.38 (0.95, 2.07)	1.46 (1.06, 2.06)	1.48 (0.98, 1.87)	0.108
FBG (mmol/L), meidan (IQR)	5.17 (4.86, 5.57)	5.36 (5.02, 5.99)	5.28 (4.96, 5.78)	<0.001
HDL (mmol/L), meidan (IQR)	1.14 (1.01, 1.33)	1.23 (1.09, 1.44)	1.26 (1.09, 1.48)	<0.001
				


**Notes.**

* *p* < 0.05, ** *p* < 0.01

HPT hypertension DBT diabetes HLP hyperlipidemia LDL low density lipoprotein TC total cholesterol TG triglyceride FBG Fiber Bragg Grating HDL high-density lipoprotein

**Table 2:** Basic clinical characteristics of patients in training set and validation set.

Characteristic	Training set				Validation set			
	No Osteopenia [*n* = 2751]	Osteopenia [*n* = 361]	Osteoporosis [*n* = 75]	*p* value	No Osteopenia [*n* = 1192]	Osteopenia [*n* = 156]	Osteoporosis [*n* = 17]	*p* value
Age (year), meidan (IQR)	38 (33, 50)	63 (55, 67)	64 (60, 67)	<0.001	38 (33, 50)	62 (55, 66)	66 (64, 67)	<0.001
Gender				<0.001				<0.001
FEMALE	1328 (41.7%)	276 (8.7%)	72 (2.3%)		576 (42.2%)	113 (8.3%)	16 (1.2%)	
MALE	1423 (44.7%)	85 (2.7%)	3 (0.1%)		616 (45.1%)	43 (3.2%)	1 (0.1%)	
Marriage				<0.001				0.333
Divorced	16 (0.5%)	1 (0%)	0 (0%)		9 (0.7%)	2 (0.1%)	0 (0%)	
Married	2620 (82.2%)	348 (10.9%)	73 (2.3%)		1125 (82.4%)	153 (11.2%)	17 (1.2%)	
Remarry	2 (0.1%)	1 (0%)	0 (0%)		2 (0.1%)	0 (0%)	0 (0%)	
Spinsterhood	108 (3.4%)	1 (0%)	0 (0%)		52 (3.8%)	0 (0%)	0 (0%)	
Widowed	5 (0.2%)	10 (0.3%)	2 (0.1%)		4 (0.3%)	1 (0.1%)	0 (0%)	
Education				<0.001				<0.001
Illiteracy	1987 (62.3%)	90 (2.8%)	11 (0.3%)		877 (64.2%)	30 (2.2%)	1 (0.1%)	
Junior high school	14 (0.4%)	14 (0.4%)	5 (0.2%)		7 (0.5%)	3 (0.2%)	2 (0.1%)	
Primary school	288 (9%)	107 (3.4%)	21 (0.7%)		109 (8%)	58 (4.2%)	6 (0.4%)	
Senior high school	58 (1.8%)	41 (1.3%)	11 (0.3%)		21 (1.5%)	22 (1.6%)	3 (0.2%)	
Higher education	404 (12.7%)	109 (3.4%)	27 (0.8%)		178 (13%)	43 (3.2%)	5 (0.4%)	
Income (yuan)				<0.001				<0.001
<1000	4 (0.1%)	13 (0.4%)	2 (0.1%)		3 (0.2%)	0 (0%)	0 (0%)	
1000–3000	18 (0.6%)	1 (0%)	1 (0%)		6 (0.4%)	1 (0.1%)	0 (0%)	
3000–5000	207 (6.5%)	79 (2.5%)	26 (0.8%)		86 (6.3%)	46 (3.4%)	8 (0.6%)	
5000–10000	2447 (76.8%)	253 (7.9%)	45 (1.4%)		1049 (76.8%)	107 (7.8%)	8 (0.6%)	
>10000	75 (2.4%)	15 (0.5%)	1 (0%)		48 (3.5%)	2 (0.1%)	1 (0.1%)	
Smoke				<0.001				0.080
NO	2084 (65.4%)	311 (9.8%)	72 (2.3%)		885 (64.8%)	129 (9.5%)	16 (1.2%)	
Quitting	81 (2.5%)	10 (0.3%)	0 (0%)		37 (2.7%)	4 (0.3%)	0 (0%)	
YES	586 (18.4%)	40 (1.3%)	3 (0.1%)		270 (19.8%)	23 (1.7%)	1 (0.1%)	
Salt(g)				0.661				0.004
<6	769 (24.1%)	110 (3.5%)	23 (0.7%)		351 (25.7%)	57 (4.2%)	7 (0.5%)	
6–12	517 (16.2%)	65 (2%)	10 (0.3%)		247 (18.1%)	15 (1.1%)	1 (0.1%)	
>12	1465 (46%)	186 (5.8%)	42 (1.3%)		594 (43.5%)	84 (6.2%)	9 (0.7%)	
Sleep (hour)				<0.001				<0.001
<4	17 (0.5%)	9 (0.3%)	3 (0.1%)		5 (0.4%)	6 (0.4%)	0 (0%)	
4–5.9	241 (7.6%)	74 (2.3%)	19 (0.6%)		91 (6.7%)	35 (2.6%)	3 (0.2%)	
6–6.9	797 (25%)	135 (4.2%)	26 (0.8%)		379 (27.8%)	65 (4.8%)	6 (0.4%)	
7–7.9	1330 (41.7%)	117 (3.7%)	23 (0.7%)		564 (41.3%)	42 (3.1%)	5 (0.4%)	
8–9.9	353 (11.1%)	22 (0.7%)	4 (0.1%)		151 (11.1%)	8 (0.6%)	3 (0.2%)	
>10	13 (0.4%)	4 (0.1%)	0 (0%)		2 (0.1%)	0 (0%)	0 (0%)	
Drink				<0.001				0.034
NO	2307 (72.4%)	325 (10.2%)	74 (2.3%)		1018 (74.6%)	144 (10.5%)	16 (1.2%)	
YES	444 (13.9%)	36 (1.1%)	1 (0%)		174 (12.7%)	12 (0.9%)	1 (0.1%)	
HPT				<0.001				<0.001
NO	2058 (64.6%)	202 (6.3%)	47 (1.5%)		902 (66.1%)	92 (6.7%)	9 (0.7%)	
YES	693 (21.7%)	159 (5%)	28 (0.9%)		290 (21.2%)	64 (4.7%)	8 (0.6%)	
DBT				<0.001				0.004
NO	2642 (82.9%)	315 (9.9%)	69 (2.2%)		1154 (84.5%)	142 (10.4%)	16 (1.2%)	
YES	109 (3.4%)	46 (1.4%)	6 (0.2%)		38 (2.8%)	14 (1%)	1 (0.1%)	
HLP				<0.001				0.003
NO	1509 (47.3%)	165 (5.2%)	29 (0.9%)		648 (47.5%)	64 (4.7%)	6 (0.4%)	
YES	1242 (39%)	196 (6.1%)	46 (1.4%)		544 (39.9%)	92 (6.7%)	11 (0.8%)	
LDL (mmol/L), meidan (IQR)	2.06 (1.59, 2.54)	2.27 (1.87, 2.81)	2.42 (1.85, 3.09)	<0.001	2.04 (1.58, 2.53)	2.41 (1.94, 2.85)	2.32 (1.8, 2.59)	<0.001
BMI (kg/m^2^), meidan (IQR)	24.3 (22, 26.6)	23.7 (21.8, 25.9)	23.2 (21.5, 25.05)	0.002	24.1 (22.1, 26.5)	23.3 (21.78, 25.13)	22.9 (21.5, 23.4)	<0.001
TC (mmol/l), meidan (IQR)	4.86 (4.28, 5.46)	5.26 (4.6, 5.93)	5.32 (4.87, 6.07)	<0.001	4.79 (4.25, 5.46)	5.33 (4.87, 6.09)	5.26 (4.5, 5.81)	<0.001
TG (mmol/l), meidan (IQR)	1.35 (0.94, 2.04)	1.43 (1.07, 2.01)	1.48 (1, 1.87)	0.094	1.43 (0.98, 2.13)	1.51 (1.02, 2.08)	1.48 (0.96, 1.79)	0.806
FBG (mmol/L), meidan (IQR)	5.17 (4.85, 5.58)	5.38 (5.03, 6.05)	5.27 (4.96, 5.68)	<0.001	5.17 (4.88, 5.56)	5.28 (5.02, 5.89)	5.6 (5.02, 5.94)	0.001
HDL (mmol/L), meidan (IQR)	1.15 (1.01, 1.33)	1.23 (1.08, 1.42)	1.26 (1.1, 1.47)	<0.001	1.14 (1, 1.32)	1.24 (1.1, 1.5)	1.14 (1, 1.48)	<0.001
								


**Notes.**

* *p* < 0.05, ** *p* < 0.01.

HPT hypertension DBT diabetes HLP hyperlipidemia LDL low density lipoprotein TC total cholesterol FBG Fiber Bragg Grating HDL high-density lipoprotein

### Identification of bone loss risk factors and construction of nomogram prediction models

**Figure 1:**
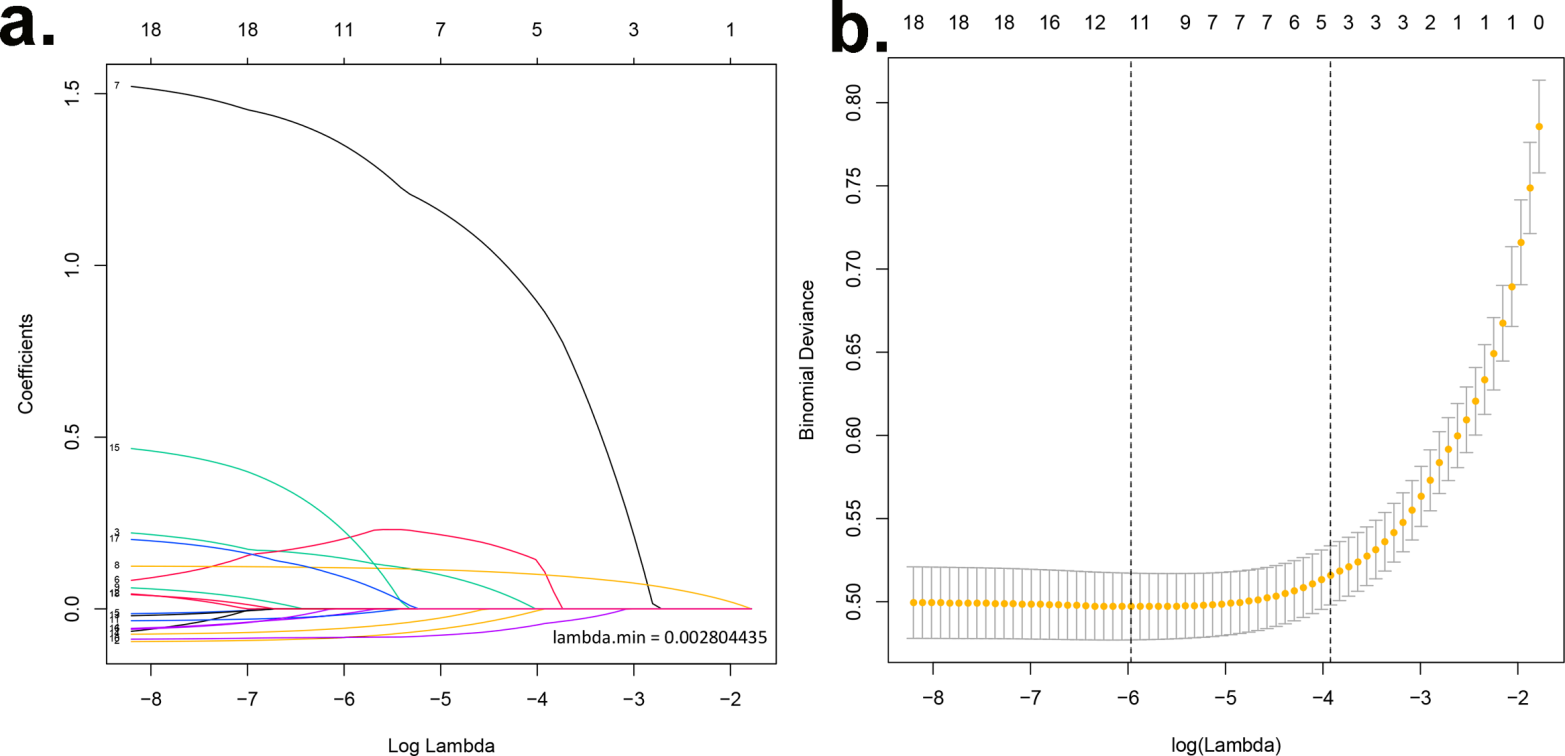
Identification of key determinants associated with bone deterioration using LASSO regression analysis. (A) The LASSO coefficient profiles of the 11 predictors. The vertical line is drawn at the optimal value by using 10-fold cross-validation *via* minimum criteria. This plot presents the profile of each coefficient against the log(lambda) sequence, where lambda represents the tuning parameter. The LASSO regression model selected 11 non-zero coefficients out of the total predictors, which include body mass index (BMI), total cholesterol (TC), triglycerides (TG), high-density lipoprotein (HDL), gender, chronological age, educational attainment, income status, sleep patterns, alcohol consumption, and diabetes mellitus. These factors have been identified as primary determinants correlated with osteal degradation. (B) Distributions of the selected predictors based on the optimal lambda. The upper panel shows the standardized coefficient of the predictors. The lower panel indicates the logarithm of the lambda value in the LASSO model. The dashed vertical lines represent the optimal lambda values that resulted in non-zero coefficients. Both panels collectively demonstrate the relative importance and contribution of each determinant in predicting osteopenia and osteoporosis.

**Figure 2:**
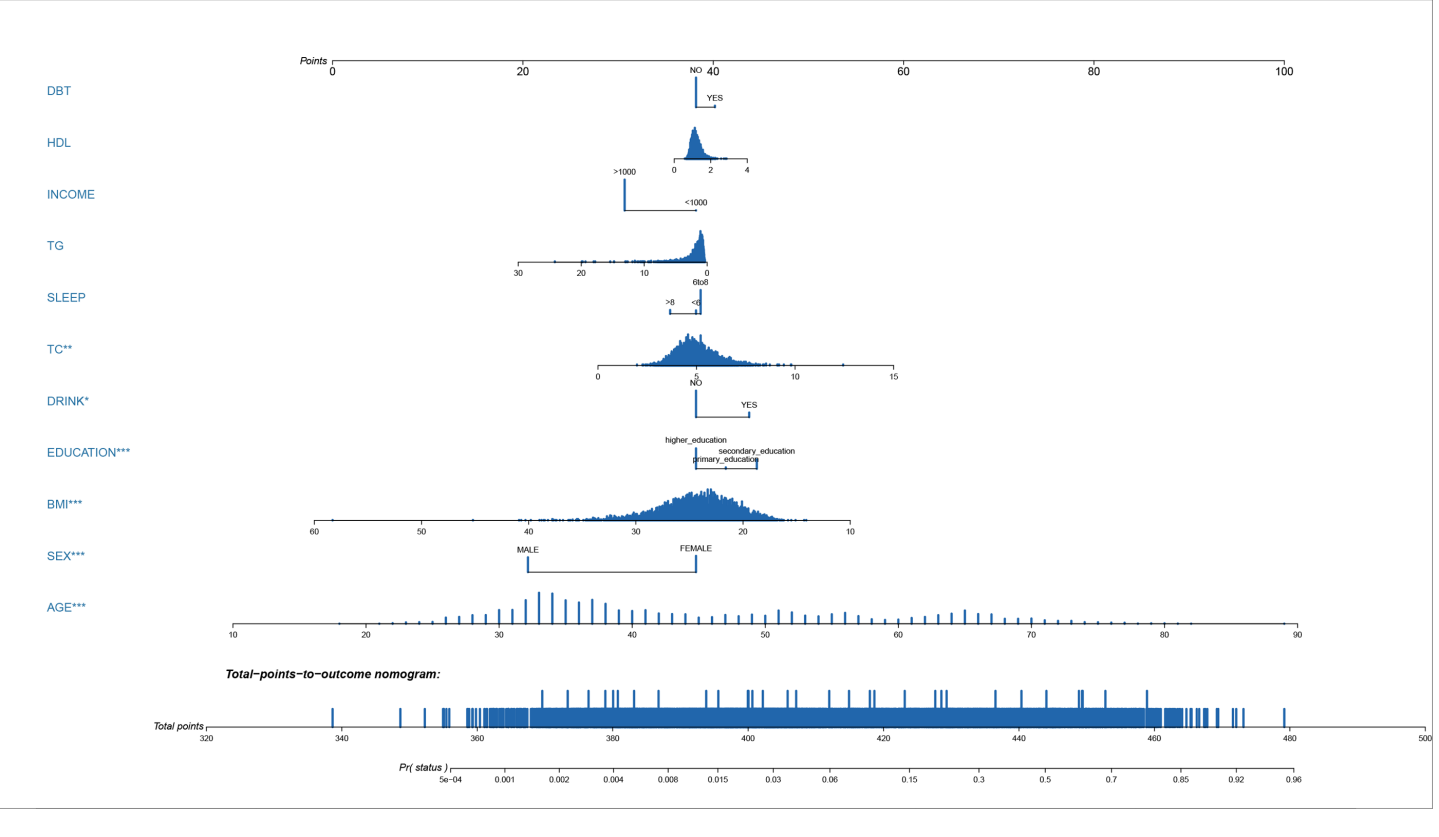
Nomogram prediction model. A nomogram prediction model for the risk of bone loss in the population.

A LASSO regression analysis discerned the principal determinants correlated with osteal diminishment, encompassing factors such as body mass index (BMI), total cholesterol (TC), triglycerides (TG), high-density lipoprotein (HDL), gender, chronological age, pedagogical attainment, financial status, nocturnal habits, alcohol indulgence, and diabetes mellitus (Figs. 1A & 1B). Based on these risk factors, we developed a nomogram to predict the risk of bone loss (Fig. 2 and Table 3). The nomogram operates by determining the score of each factor on the designated axis, summing these individual scores to give a total score, and then reading the corresponding risk value for bone loss from the nomogram. This process allows for individualized risk prediction for each patient.

**Table 3:** Prediction factors for osteopenia using multivariable logistic regression analysis.

Variable	Prediction model		
	*β*	Odds ratio (95% CI)	*P*-value
(Intercept)	−4.726	0.907(0.873 ∼0.942)	*p* < 0.001
BMI	−0.097	0.907(0.873 ∼0.942)	*p* < 0.001
TC	0.179	1.196(1.049 ∼1.364)	0.007
TG	−0.058	0.944(0.855 ∼1.041)	0.248
HDL	0.171	1.186(0.715 ∼1.966)	0.509
Gender	−1.533	0.216(0.162 ∼0.288)	*p* < 0.001
Age	0.122	1.129(1.117 ∼1.142)	*p* < 0.001
Education	0.279	1.322(1.162 ∼1.504)	*p* < 0.001
Income	−0.648	0.523(0.195 ∼1.406)	0.199
Sleep	−0.108	0.898(0.758 ∼1.063)	0.210
Drink	0.486	1.626(1.079 ∼2.449)	0.020
Diabetes	0.169	1.184(0.813 ∼1.725)	0.380

**Notes.**

*β* is the regression coefficient.

### Assessment of the nomogram model’s predictive accuracy

**Figure 3:**
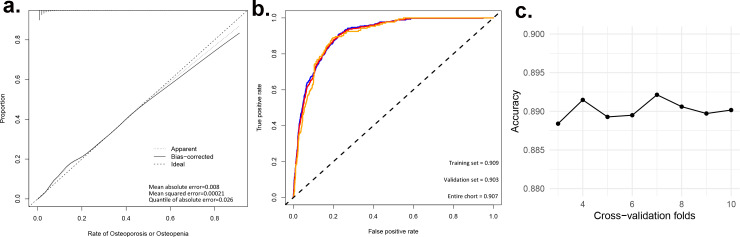
Evaluating the predictive power of nomogram models. (A) Predictive models for the risk of bone loss in the population; (B) ROC and AUC for the risk of bone loss in the population; (c) Accuracy of logistic regression model for different k-fold cross-validation.

**Figure 4:**
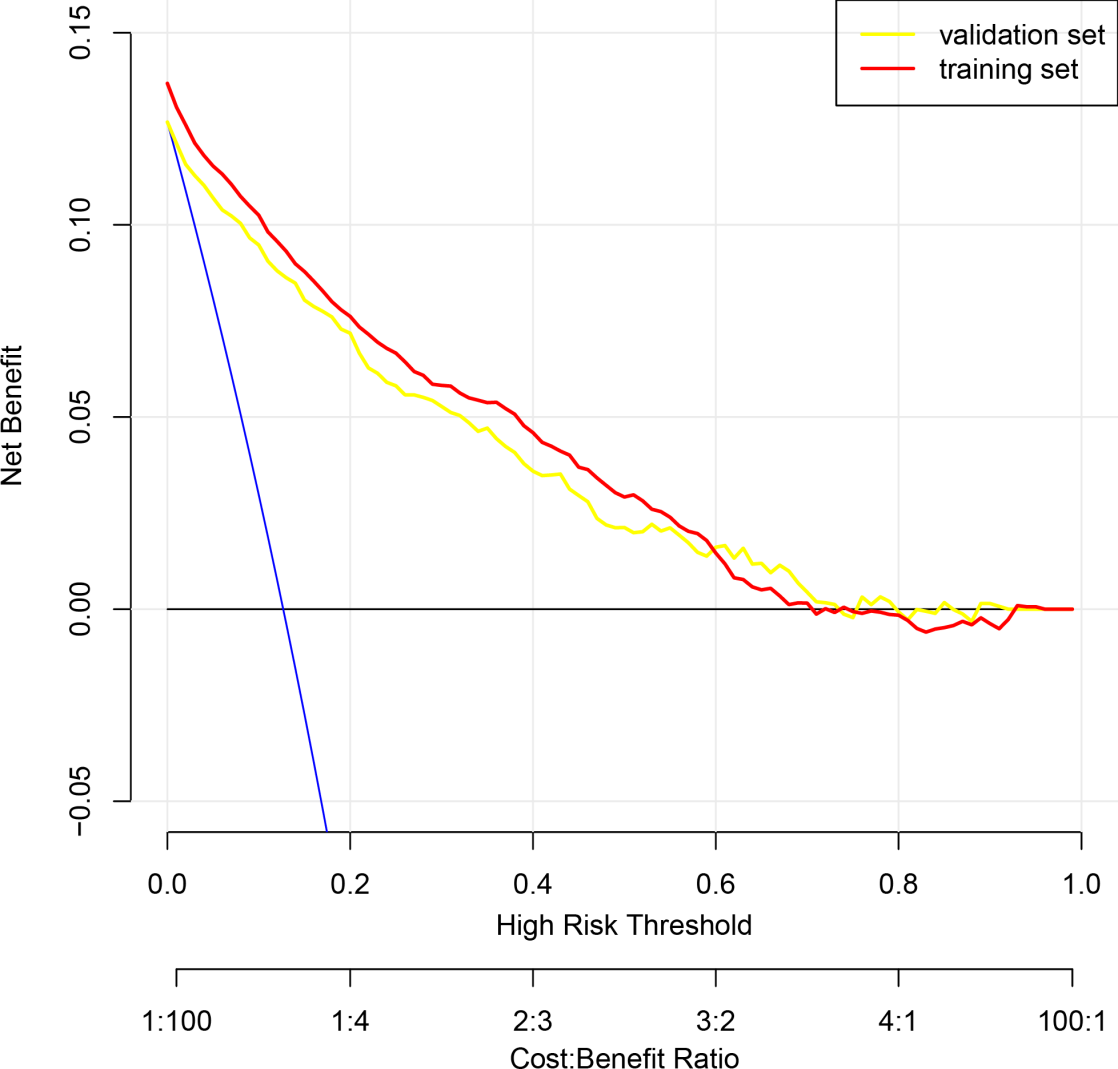
Decision curve analysis illustrating the clinical utility of the prognostic nomogram for predicting the risk of bone loss. The *y*-axis measures the net benefit derived from the use of our nomogram. The *x*-axis represents the threshold probability, which is the probability at which a patient would opt for a preventative or therapeutic measure for bone loss. The blue line indicates the nomogram. The blue line denotes the assumption that all patients will develop bone loss, whereas the black line represents the assumption that no patients will experience bone loss.

To assess the discriminatory power of our nomogram model, we computed the concordance index (C-index) for both the training set (C-index = 0.908), validation set (C-index = 0.908), and for the entire cohort(C-index = 0.910). These high C-index values underscore the nomogram’s robust discriminatory ability. To augment the veracity of our model’s efficacy, we executed cross-validation extending from a three-fold up to a ten-fold schema, alongside bootstrap validation, congruent with the advisories delineated by Iasonos et al. (2008). These additional validation steps are crucial in verifying the reliability of the nomogram model, and the results are reported in Table 4. Figure 3A presents calibration plots that depict the agreement between predicted probabilities of bone loss and observed outcomes in our study population. The close alignment of the actual curve with the standard and corrected curves signifies the accuracy of our nomogram model. We also assessed the area under the receiver operating characteristic curve (ROC) for the nomogram, achieving AUC values of 0.909, 0.903 for the training set, and the validation set respectively, further attesting to the model’s high discriminatory ability (Fig. 3B). The logistic regression model underwent rigorous cross-validation, encompassing 3-fold through to 10-fold. Remarkably, the model’s accuracy consistently hovered around 0.89 under all scenarios, demonstrating a steadfast predictive capacity. Among these, the 5-fold cross-validation emerged superior, achieving the pinnacle of accuracy at approximately 0.8915, while the 4-fold cross-validation rendered the least accuracy, approximately at 0.8893. These findings subtly indicate that the number of folds does impart an influence on the model’s accuracy, albeit relatively minimal (Fig. 3C). Lastly, decision curve analysis was performed, demonstrating that the nomogram’s net benefit curve was above the all-benefit and no-benefit reference lines. This indicates that our nomogram can provide a beneficial prediction of the risk of bone loss in patients, substantiating its clinical applicability (Fig. 4). In summary, our model exhibits impressive predictive prowess and stability.

**Table 4:** C-index of the prediction model.

Dataset group	C-index of the prediction model	
	C-index	The C-index (95% CI)
Training set	0.908	0.898–0.919
Validation set	0.908	0.895–0.921
Entire Cohort	0.91	0.891–0.929

## Discussion

Osteoporosis, a major skeletal disease marked by diminished bone density and damage to the bone tissue’s microarchitecture, significantly increases the risk of fractures (Faulkner et al., 1993). These fractures, termed osteoporotic fractures, impose a substantial burden on both the individual and the healthcare system (Clynes et al., 2020). To address this, there is an urgent need for precise, accessible, and easy-to-use tools that can predict the risk of osteoporosis based on clinical variables. This is crucial for initiating early interventions, thus helping to reduce the risk of fractures and the associated societal and healthcare burdens. Herein lies the potential of our nomogram prediction model, which has been designed to meet these very clinical needs. In recent years, machine learning has been making inroads into the medical field, including the prediction of osteoporotic fractures (Kilic & Hosgormez, 2016; Kruse, Eiken & Vestergaard, 2017; Wang et al., 2019; Villamor et al., 2020). Nevertheless, there are concerns about the precision and practicality of these methods in a clinical setting, factors our nomogram model addresses with its high accuracy and ease of use.

In this study, a risk prediction model for bone loss was developed. Drawing from a substantial sample of 4,552 cases, our study demonstrated a higher predictive power than previous studies (Wang et al., 2021). Eleven indicators, such as BMI, TC, TG, HDL, Gender, Age, Education, Income, Sleep, Alcohol Consumption, and Diabetes, were identified as key risk factors associated with bone loss. By understanding how each of these factors impact bone health, our nomogram prediction model was constructed, which can facilitate early detection and intervention. This is vital as the model enables clinicians to estimate patient prognosis and risk stratification more accurately, thereby informing treatment planning and decision-making. Furthermore, it aids in patient counseling, enabling patients to comprehend their prognosis better and make informed choices about their treatment options. In addition, our model’s identification of key prognostic factors could guide future research aimed at developing innovative therapeutic strategies targeting these factors.

While genetic factors account for 60% to 90% of the variation in human bone mass, we understand the importance of shedding more light on the contribution of external factors such as living environment, physical activity, nutritional status, age, and gender to bone health (Landin-Wilhelmsen, Wilhelmsen & Bengtsson, 1999). Studies have shown that bone loss occurs more rapidly and is more pronounced in women than in men, with the rate of bone loss after menopause reaching 2.2% to 3.0% per year. In fact, the total bone loss rate in women can reach 20% to 30% during the 20 years post-menopause (Li et al., 2002). Therefore, it becomes crucial for women to initiate preventative measures against osteoporosis as early as possible before menopause. In contrast, the incidence of osteoporosis is highest in menopausal women, but is expected to triple in men in the coming decades (Gullberg, Johnell & Kanis, 1997). Osteoporosis tends to increase with age, and the bone structure defects caused by it are irreversible. Thus, early detection of bone loss and maximizing peak bone mass have emerged as vital preventative measures against osteoporotic fractures (Bonura, 2009; Kling, Clarke & Sandhu, 2014).

Recognizing the significance of discerning risk factors and comprehending their influence on skeletal well-being, we have considerably augmented our discourse segment in the manuscript to encompass a more comprehensive scrutiny of how each ascertained risk factor impacts bone health. This has been particularly expounded within the framework of diabetic osteoporosis, a systemic, metabolic bone ailment. This condition has surfaced as a prevalent complication gravely compromising the quality of life in elderly diabetic patients (Paschou et al., 2017; Johnston & Dagar, 2020). Moreover, with a surge in the diagnosed cases of diabetes, diabetic osteoporosis has become a prevailing complication, underscoring a profound correlation between these two conditions (Ala, Jafari & Dehpour, 2020). Our study findings propose that 31.3% of patients with type 2 diabetes exhibit diminished bone mass, thereby indicating a significantly heightened risk of osteoporosis in this population. Lipid, being one of the most vital energy metabolites in the human body, and its metabolic disorders can incite diverse maladies, such as hypercholesterolemia, obesity, arterial sclerosis, hepatic steatosis, hypertension, and so forth (Ertunc & Hotamisligil, 2016). The burgeoning attention on maladies associated with aberrant lipid metabolism and osteoporosis in recent years (Ertunc & Hotamisligil, 2016). Accentuating the close association between the bone microenvironment and bone health, we delve into how bone loss and osteoporotic fractures can manifest concomitantly in patients with hypercholesterolemia (Luo et al., 2021). Additionally, osteoporosis and diminished bone mass are characterized by anomalous lipid metabolism and vascular calcification (Hu et al., 2019). Intricate processes such as the differentiation of adipocytes and osteoblasts from bone marrow stem cells, as well as the potential impact of a chronic high-fat diet on facilitating adipogenesis, impeding osteogenesis, and augmenting the risk of osteoporosis, are also addressed (Hu et al., 2018). Furthermore, we delve into the phenomenon whereby bone marrow osteoblasts tend to transdifferentiate into adipocytes, a process potentially instigated by the intrinsic properties of adipocytes themselves (Paspaliaris & Kolios, 2019). In accordance with this, our study has identified total cholesterol, triglycerides, and high-density lipoprotein as risk factors for bone loss.

In line with our commitment to a comprehensive identification and analysis of risk factors affecting skeletal health, we have augmented our discussion section with a thorough analysis of each confirmed risk factor and its potential impact on bone health. These risk factors include sleep duration and education level. A growing body of research establishes a relationship between sleep duration and osteoporosis, suggesting that both excessive and insufficient sleep duration can impact bone density. In a cross-sectional study evaluating the link between sleep duration and osteoporosis in postmenopausal women, Ochs-Balcom et al. (2020) discovered that women sleeping no more than 5 h per night were at a higher risk of developing low bone mass and osteoporosis compared to those who slept 7 h per night (Moradi et al., 2017; Ochs-Balcom et al., 2020). Meanwhile, a meta-analysis probing into the relationship between sleep duration and osteoporosis in middle-aged and elderly individuals found a U-shaped correlation, with the lowest risk associated with approximately 8 h of sleep per day (Wang et al., 2018). This indicates that both excessive and insufficient sleep can elevate the risk of osteoporosis (Lucassen et al., 2017; Wang et al., 2018). Moreover, the education level appears to be a significant factor as well. Individuals with a higher education level are generally linked with improved health awareness, healthier behaviors, better socio-economic status, living conditions, and social well-being (Brennan-Olsen et al., 2015; El Hage et al., 2019). In our study, we found a correlation between literacy and the risk of bone loss.

Future studies should aim for advancements in several areas: (i) Our study considered a select sample of characteristics, which may inadvertently introduce bias.; (ii) Further validation of the accuracy and reliability of the nomogram is necessary through prospective, multiethnic, and multicenter studies. These future studies should not only confirm our findings but also explore the potential molecular mechanisms underlying the observed associations. Additionally, assessing potential treatment targets and interventions based on our findings may lead to new treatment strategies for the specific conditions studied in our manuscrip. Collaborations with other research teams for meta-analyses and pooled analyses will also help strengthen the evidence supporting our conclusions. Our study has potential limitations, including sample size, the use of animal models, and potential confounding factors that can influence outcomes. It is crucial to interpret our findings with caution, recognizing the necessity for further research to confirm our discoveries. We also acknowledge the limitations of our study population and the need for further research in different populations to confirm and expand our findings. Potential health issues related to skeletal health, such as vitamin D deficiency, hormonal imbalance, or other chronic diseases, were not considered. Nevertheless, our study, with a sample of more than 4,000 cases, resulted in a high confidence predictive model. This provides reliable evidence to support the development of rational and scientific treatment plans for patients and the initial assessment of the risk of bone loss in the population.

## Conclusion

In conclusion, our nomogram, based on basic population characteristics (including BMI, TC, TG, HDL, Gender, Age, Education, Income, Sleep, Drink, and Diabetes), can help predict the risk of bone loss in a population. It is expected to assist people in becoming aware of the risk of bone loss and respond accordingly. This study provides reliable evidence to support the development of rational and scientific treatment plans for patients and initial assessment of the risk of bone loss in the population. Yet, further validation of the accuracy and reliability of the nomogram is needed in later prospective, multiethnic, and multicenter studies.

##  Supplemental Information

10.7717/peerj.16017/supp-1 Data S1 Raw data

10.7717/peerj.16017/supp-2 Figure S1 Correlation heatmap of bio-social parameters in a population sample. The pearson correlation coefficients between the variables, with a color gradient ranging from blue (negative correlation) to red (positive correlation). A value closer to +1 or −1 indicates stronger correlation.

## Additional Information and Declarations

### Data Availability

The following information was supplied regarding data availability: All raw data are available in the Supplemental File.
