# Supplementary figures and images for: Retrospective study: risk assessment model for osteoporosis—a detailed exploration involving 4,552 Shanghai dwellers
Source: PeerJ. 2023 Sep 8;11:e16017. doi: 10.7717/peerj.16017 (PMC10494836; doi:10.7717/peerj.16017)

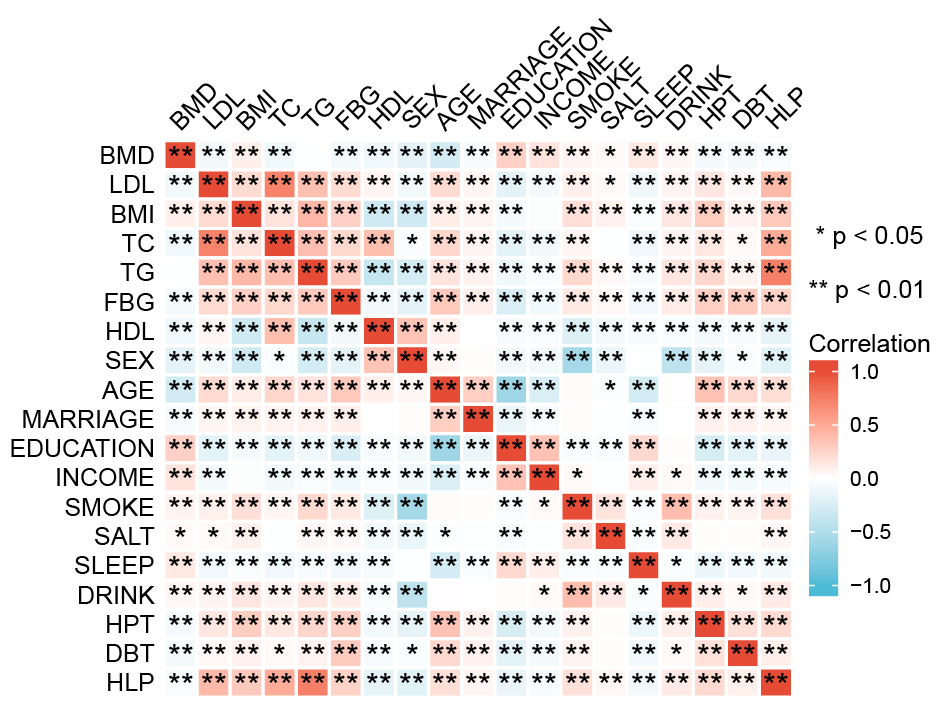

Supplement: Figure S1 — The pearson correlation coefficients between the variables, with a color gradient ranging from blue (negative correlation) to red (positive correlation). A value closer to +1 or −1 indicates stronger correlation. [file peerj-11-16017-s002.png]
